# Supplementary material for: Oral Administration of Artemisone for the Treatment of Schistosomiasis: Formulation Challenges and In Vivo Efficacy
Source: Pharmaceutics. 2020 Jun 3;12(6):509. doi: 10.3390/pharmaceutics12060509 (PMC7356104; doi:10.3390/pharmaceutics12060509)
Supplement: Supplementary file 1 [file pharmaceutics-12-00509-s001.pdf]

pharmaceutics

MDPI

Supplementary Materials: Oral Administration of Artemisone for the Treatment of Schistosomiasis: Formulation Challenges and In Vivo Efficacy

Johanna Zech, Daniel Gold, Nadeen Salaymeh, Netanel Cohen Sasson, Ithai Rabinowitch, Jacob Golenser and Karsten Mäder

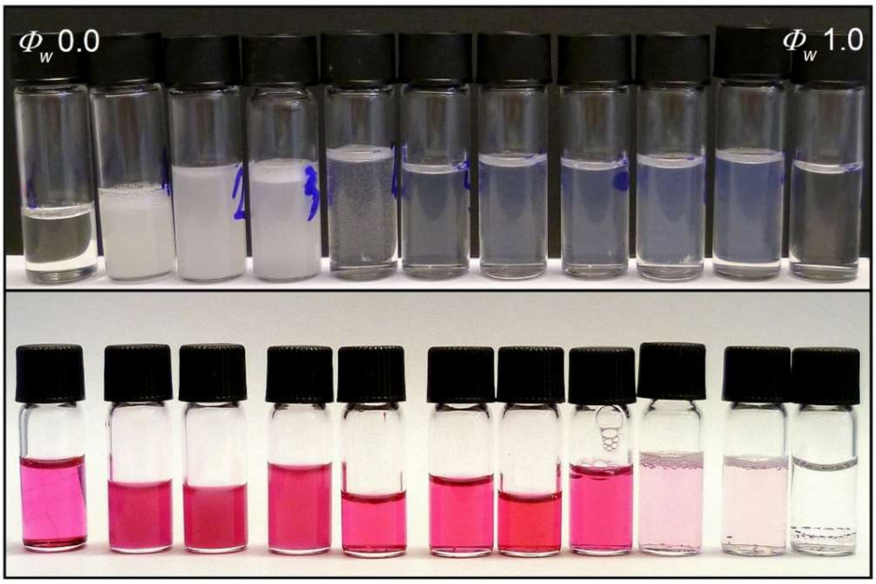

Figure S1. Impact of PBS content on SMEDDS properties at 25 °C. **A**: after mixing of different ratios between SMEDDS and PBS, turbidity of formulations with a PBS content of 10–30%indicated the formation of a coarse water-in-oil emulsion. All other formulations were transparent or bluish opalescent. **B**: Results of the solubilization assay: Sudan Red powder was placed in the vial before adding the formulation. Formulations with a water content of ≤ 70 % were able to dissolve Sudan Red, indicating the presence of a percolating lipophilic phase.

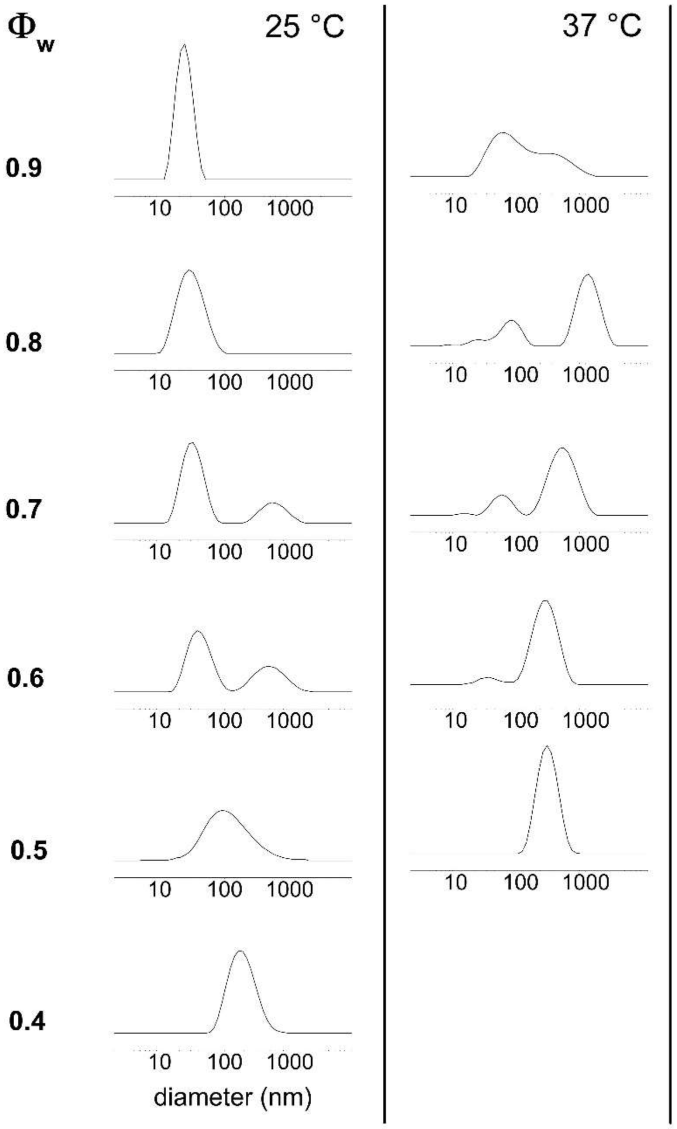

Figure S2. Impact of composition and temperature on particles size distribution (intensity weighted dynamic light scattering). The measurement of the formulation SMEDDS-PBS with a water content of 0.4 at 37 °C was not possible because the particle size was too large and particle sized distribution polydisperse.

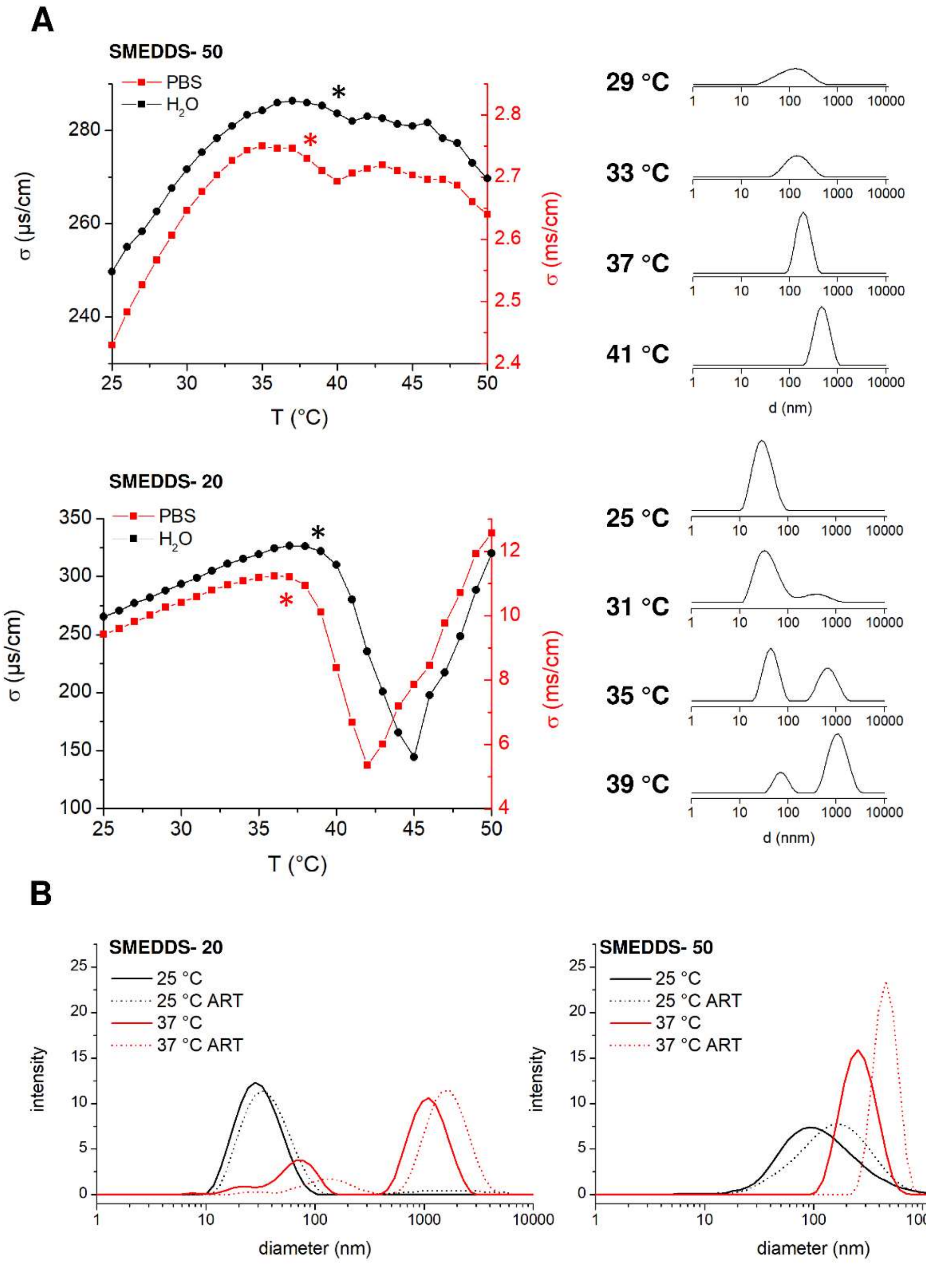

Figure S3. A. Impact of temperature on conductivity and particle size (intensity distribution, dynamic light scattering) on SMEDDS-50 and SMEDDS-20 formulations. The cloud point is indicated by* B. Impact of drug load and temperature on particle diameter as obtained via dynamic light scattering.

A

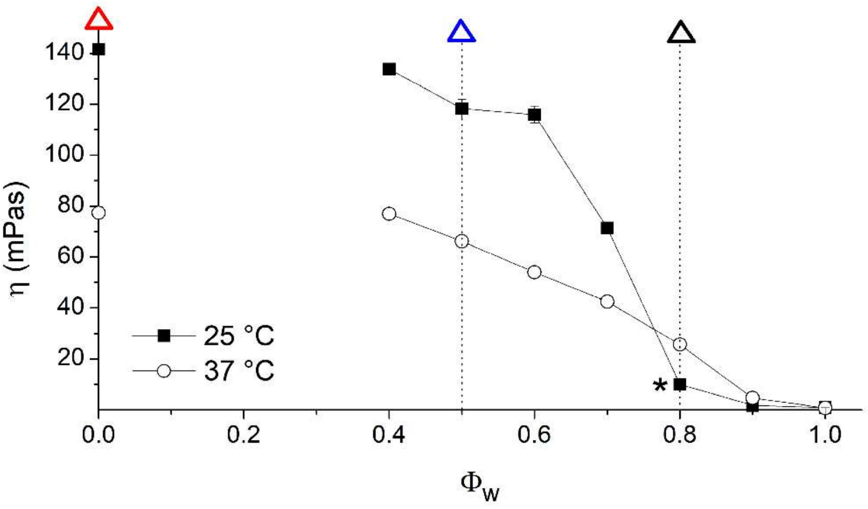

B

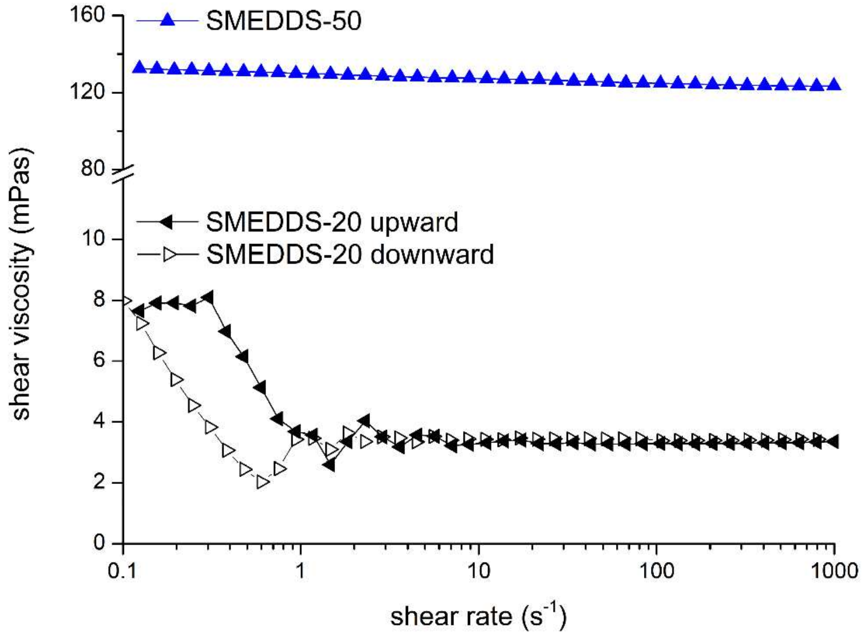

Figure S4. Impact composition and temperature on the dynamic viscosity measured with a capillary viscometer (**A**) and shear viscosity of SMEDDS-50 and SMEDDS-20 analyzed with a rotational rheometer (**B**).

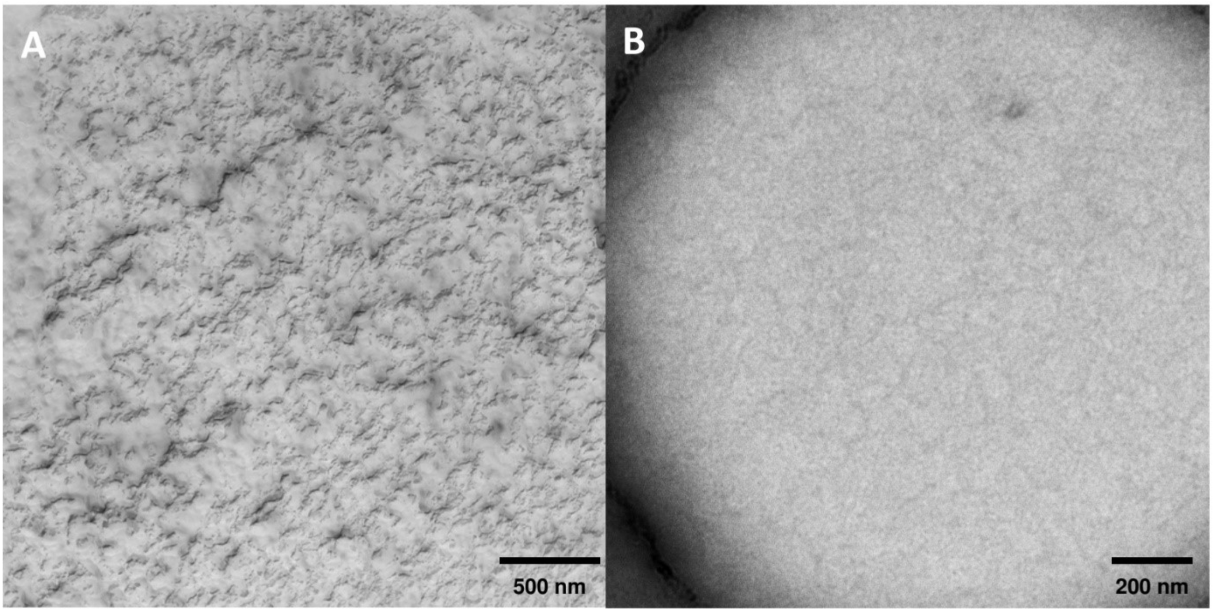

Figure S5. Freeze fracture micrographs of SMEDDS-50 (**A**) and SMEDDS-20 (**B**). The pictures indicate a bicontinuous structure for SMEDDS-50 and cylindrical micelles for SMEDDS-20.

Table S1. Effect of Salinity; Cloud points for SMEDDS-20 and SMEDDS-50 formed with distilled water, 1-/5-/10-fold PBS.

Salinity (g/kg)	Cloud point (°C)	
	SMEDDS-50	SMEDDS-20
0.0 (H_2O)	40.0	37.5
9.6 (1-fold PBS)	38.0	36.0
48.0 (5-fold PBS)	29.5	35.0
96.0 (10-fold PBS)	< 20	29.0

© 2020 by the authors. Submitted for possible open access publication under the terms and conditions of the Creative Commons Attribution (CC BY) license (http://creativecommons.org/licenses/by/4.0/).
